# Flash Pulmonary Oedema in a Patient With Unilateral Renal Artery Stenosis and Preserved Contralateral Function: A Medically Managed Case Report

**DOI:** 10.7759/cureus.98005

**Published:** 2025-11-28

**Authors:** Mohamad Abu Zaher, Ching Lee, Sumith Abeygunasekar

**Affiliations:** 1 Nephrology, Broomfield Hospital, Chelmsford, GBR

**Keywords:** cardiorenal syndrome, flash pulmonary oedema, pickering phenotype, renal artery stenosis, renovascular hypertension

## Abstract

Flash pulmonary oedema (Pickering phenotype) is a high-risk, under-recognised manifestation of renovascular disease. A man in his 50s presented with abrupt dyspnoea, hypertensive crisis, type 1 respiratory failure, and pulmonary oedema. His response to intravenous diuretics was limited, and he was stabilised in the ICU with glyceryl trinitrate and continuous positive airway pressure. Ultrasound showed a small scarred right kidney of 7.9 cm. Echocardiography found a left ventricular ejection fraction (LVEF) of 25% at presentation. Three months later, cardiac MRI showed LVEF of 40% with diffuse mid-wall scarring. Renal MR angiography (MRA) confirmed severe ostial right renal artery stenosis, and dimercaptosuccinic acid (DMSA) demonstrated a split kidney function of 36% in the right and 64% in the left. A medical-first strategy was chosen after multidisciplinary discussion, with no recurrent episodes of pulmonary oedema during follow-up. This case illustrates the difficulty of attributing flash pulmonary oedema to unilateral atherosclerotic renovascular disease in the context of long-standing hypertensive heart and kidney disease and highlights how laterality, kidney size, and differential function can guide the choice between selective revascularisation and conservative management.

## Introduction

Renal artery stenosis (RAS) is a modifiable, renovascular cause of malignant hypertension and chronic kidney disease (CKD). It can present as flash pulmonary oedema (Pickering phenotype) [1,2]. Renal hypoperfusion across a tight lesion activates the renin-angiotensin-aldosterone system (RAAS), which drives salt and water retention and vasoconstriction. In patients with diastolic dysfunction, small volume shifts trigger a significant rise in filling pressure and pulmonary oedema [1,3]. Classically, this presentation occurs with bilateral RAS or unilateral RAS in a solitary functioning kidney [2]. We report acute flash pulmonary oedema occurring in a patient with a unilateral right RAS in a small, hypofunctioning kidney and a normal left kidney. In addition, we outline a conservative, medical-first management strategy without invasive revascularisation.

Acute pulmonary oedema is most often precipitated by acute left ventricular systolic or diastolic dysfunction, myocardial ischaemia, arrhythmias, valvular disease, or sudden rises in blood pressure leading to increased afterload [4,5]. In patients with long-standing hypertension and chronic kidney disease, it can be difficult to determine whether decompensation is driven predominantly by primary cardiac pathology, haemodynamic and volume shifts related to kidney disease, or a combination of both [4]. Renovascular disease, particularly bilateral RAS or stenosis affecting a solitary functioning kidney, is a recognised cause of recurrent “flash” pulmonary oedema (Pickering phenotype), whereas the clinical significance of unilateral RAS with preserved contralateral function is less well defined [5,6].

## Case presentation

A man in his 50s presented to the emergency department with acute breathlessness and a productive cough. He reported two weeks of progressively worsening exertional dyspnoea and reduced exercise tolerance. His past medical history included hypertension and chronic kidney disease, with a baseline creatinine of 165 µmol/L (60-110 µmol/L). He was an active smoker, smoking five cigarettes per day. He was followed up by his general practitioner and was taking regular amlodipine for hypertension. He had no previous hospital admissions.

**Figure 1:**
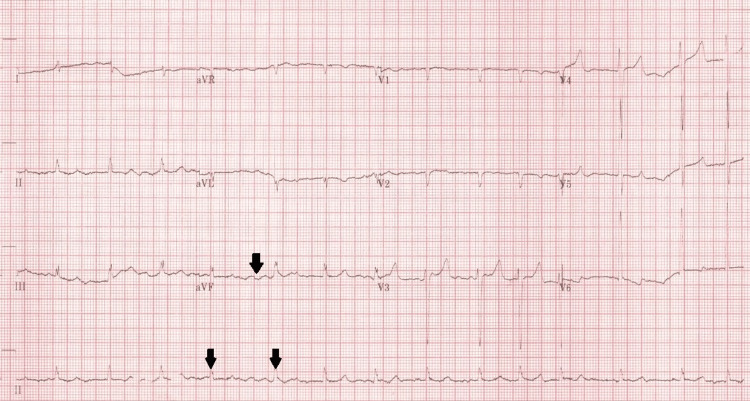
Atrial fibrillation on 12-lead ECG Twelve-lead ECG demonstrating atrial fibrillation with an irregularly irregular ventricular response of approximately 100 beats per minute. Arrows highlight the irregular R–R intervals and absence of organised P-waves.

**Figure 2:**
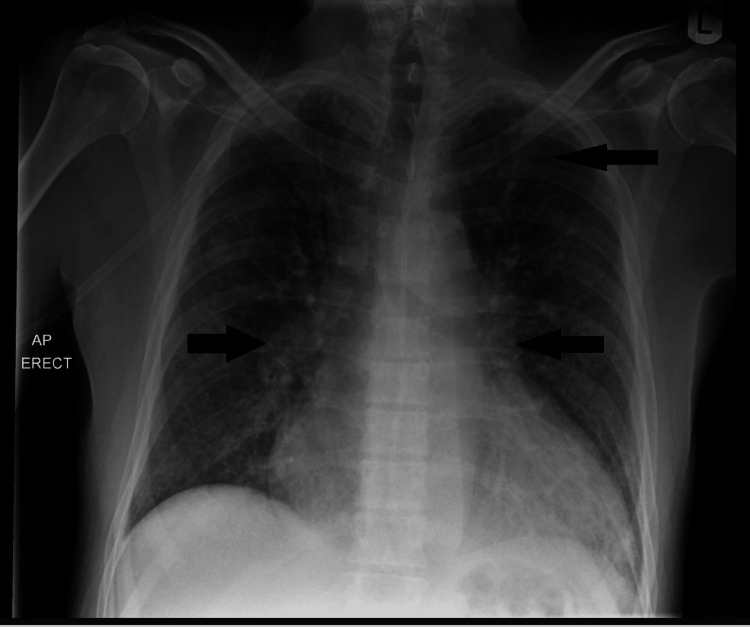
Chest radiograph demonstrating pulmonary oedema Anteroposterior chest radiograph demonstrating pulmonary vascular congestion and bilateral perihilar pulmonary oedema highlighted by the arrows.

He was acutely unwell in the emergency department with respiratory distress, hypoxia requiring Venturi oxygen, tachycardia, and severe hypertension at 210/120 mmHg. At his worst, his respiratory rate was 40 breaths per minute. Examination revealed raised jugular venous pressure, bilateral pitting oedema to the feet, and bilateral lung crackles. The ECG showed atrial fibrillation, with a ventricular rate of 100-120 bpm (Figure 1). The chest radiograph demonstrated features of pulmonary vascular congestion and bilateral perihilar pulmonary oedema (Figure 2). An arterial blood gas confirmed type 1 respiratory failure. Initial blood tests showed creatinine 260 µmol/L (60-110 µmol/L) and urea 20.6 mmol/L(2.5-7.8 mmol/L), with normal inflammatory markers. NT-proBNP (N-Terminal Pro-B-type Natriuretic Peptide) was 14,400 ng/L (<400 ng/L).

He was treated for acute pulmonary oedema precipitated by new-onset heart failure, with acute-on-chronic kidney injury. Despite starting an intravenous furosemide infusion, there was little initial improvement, and his care was escalated to the intensive care unit within four hours of arrival. In the ICU, he received an intravenous glyceryl trinitrate infusion and continuous positive airway pressure, with prompt improvement in his work of breathing and oxygenation. He was subsequently stabilised and stepped down to the medical ward.

**Figure 3:**
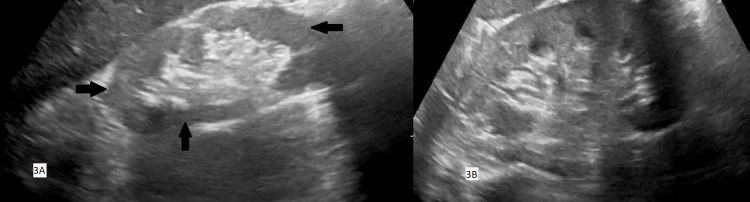
Renal ultrasound demonstrating asymmetry between kidneys (A) Right kidney ultrasound demonstrating cortical thinning, decreased corticomedullary differentiation, and increased echogenicity consistent with chronic scarring as shown by the arrows. (B) Left kidney ultrasound showing normal cortical thickness and echotexture.

His renal autoimmune screen was negative, with a 5 mg/mmol urine albumin-creatinine ratio (<3 mg/mmol). Renal ultrasound showed a small right kidney measuring 7.9 cm with cortical scarring (Figure 3, Panel A). The left kidney was of normal size (10 cm) and echotexture, with no hydronephrosis (Figure 3, Panel B). Given the kidney size asymmetry in the context of a hypertensive episode with flash pulmonary oedema, he was scheduled for an outpatient renal MRA to evaluate for RAS.

A formal inpatient echocardiogram confirmed acute heart failure with a left ventricular ejection fraction of 25%, with the heart failure team comanaging his care. After stabilisation, he was discharged following a two-week admission on eplerenone, dapagliflozin, edoxaban, and bisoprolol, with outpatient follow-up arranged for optimisation and monitoring of renal function and electrolytes.

At three months, his creatinine remained stable at 260-270 µmol/L(60-110 µmol/L). Cardiac MRI showed improvement in left ventricular ejection fraction to 40%, from 25%, with diffuse mid-wall scarring, consistent with a hypokinetic, non-dilated left ventricular cardiomyopathy. He was reviewed in the cardiology clinic, where long-standing hypertension was considered a likely contributor to the initial decompensation. Follow-up continued, with optimisation of medical therapy and surveillance of renal function and electrolytes.

**Figure 4:**
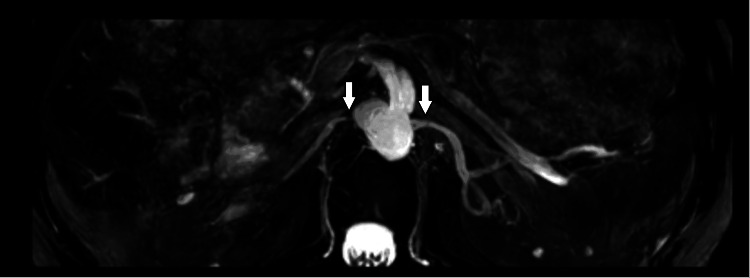
Contrast-enhanced MRA (coronal MIP) demonstrating severe ostial stenosis of the right renal artery MR angiography (MRA) showing focal stenosis of the right renal artery (arrows). MIP: Maximum intensity projection.

**Figure 5:**
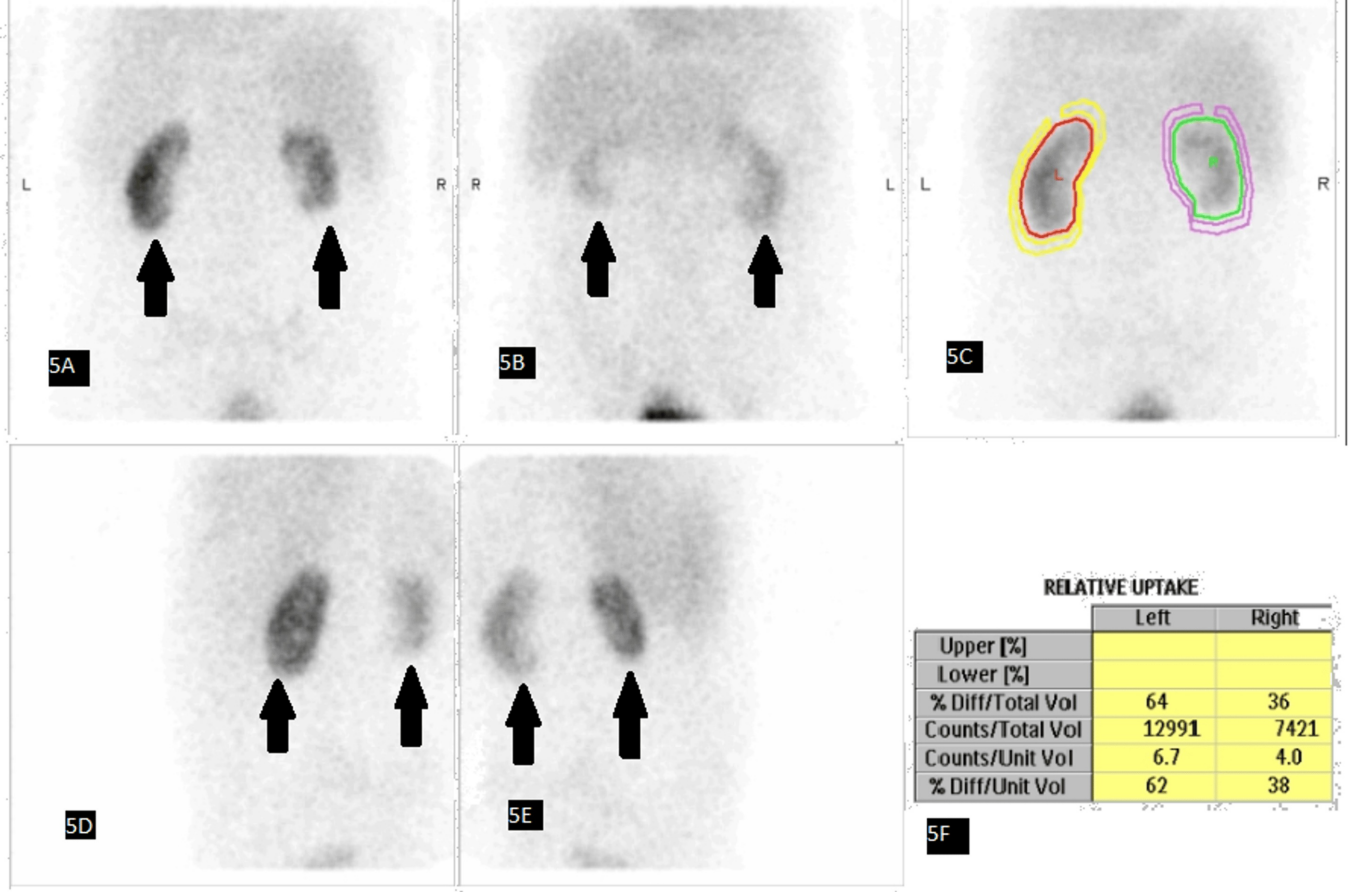
DMSA demonstrated a split function of 36% right/64% left (A) Posterior DMSA projection demonstrating reduced tracer uptake in the right kidney as shown by the arrows. (B) Anterior projection again demonstrating relatively reduced right renal uptake as shown by the arrows. (C) Geometric region-of-interest (ROI) analysis outlining the left and right kidneys. (D) Left posterior oblique projection demonstrating reduced tracer uptake in the right kidney as shown by the arrows. (E) Right posterior oblique projection demonstrating reduced tracer uptake in the right kidney as shown by the arrows. (F) Relative renal uptake table demonstrating asymmetric differential function (left 64%, right 36%). DMSA: Dimercapto succinic acid.

Renal MRA confirmed severe proximal (ostial) stenosis of the right renal artery (Figure 4). The distal right renal artery and the entire left renal artery were unobstructed. Dimercaptosuccinic acid (DMSA) renal scintigraphy demonstrated a split renal function of 36% in the right kidney and 64% in the left kidney (Figure 5). In the renal clinic, he was counselled about management options, including endovascular revascularisation against continued medical therapy. The discussion covered that the left kidney is functioning well, and the right kidney, although small and hypofunctioning, is not nonfunctioning, so the expected renal salvage from stenting would likely be limited. After shared decision-making and review of the risks and benefits of intervention, he elected for a medical-first approach focused on strict blood pressure control, smoking reduction, and avoidance of nephrotoxic agents. Follow-up was arranged to monitor renal function, blood pressure, and symptoms, with revascularisation to be reconsidered if decompensation recurred or control proved suboptimal.

The cardiology and nephrology teams jointly optimised his medications to control blood pressure, prevent further renal decline, and avoid fluid overload. He was prescribed hydralazine, isosorbide mononitrate, carvedilol, dapagliflozin, atorvastatin, and rivaroxaban. Follow-up was arranged in three months with repeat renal function, electrolytes, and blood pressure review.

## Discussion

Pathophysiology

RAS is a luminal narrowing of the main renal artery or its major branches [7]. Most often atherosclerotic in nature, it usually occurs in the ostium or proximal third of the artery, leading to renal hypoperfusion, which may contribute to flash pulmonary oedema (Pickering phenotype) in susceptible patients [7]. When pressure distal to a lesion falls, the RAAS is triggered, causing salt and water retention, which increases preload, additionally causing vasoconstriction, which increases afterload. In patients with left ventricular hypertrophy or diastolic dysfunction, even small volume shifts produce disproportionate rises in left ventricular filling pressure, which can precipitate flash pulmonary oedema [3,7]. Classically, this is volume-dependent in bilateral RAS or unilateral RAS with a solitary functioning kidney because there is no contralateral natriuresis [3]. In unilateral RAS with two kidneys, oedema is uncommon but documented when ventricular compliance is reduced, or the stenosed kidney is small or hypofunctioning, and rapid improvement has been reported after revascularisation in such atypical cases [8]. Haemodynamically significant RAS is often defined as at least 70% diameter stenosis or 50%-70% plus a peak gradient greater than 20 mmHg or a mean gradient of at least 10 mmHg on a pressure wire. These thresholds align with the underlying physiology and guide intervention in the flash pulmonary oedema phenotype [7].

Under-recognition

Despite clear pathophysiology, renovascular causes of flash pulmonary oedema are often under-investigated. Contemporary reviews highlight that major revascularisation trials in broad RAS cohorts (e.g., angioplasty and stenting for renal artery lesions (ASTRAL) and cardiovascular outcomes in renal atherosclerotic lesions (CORAL)) enrolled many patients without haemodynamically severe disease and did not include the flash oedema phenotype, yielding neutral results that were then over-generalised to all RAS presentations [2,9,10]. This contributed to a marked decline in renal arteriography and case findings, while patients with Pickering phenotype were managed as generic heart failure, in whom RAAS blockade may worsen renal function [2].

Red flags that should trigger an RAS workup

A renovascular cause should be suspected when acute or recurrent flash pulmonary oedema occurs with severe or resistant hypertension, when previously stable blood pressure deteriorates abruptly, or when there is a rise in creatinine after starting an angiotensin-converting enzyme (ACE) inhibitor or angiotensin receptor blockers (ARBs) [1,11]. Classically, around a 30% increase in creatinine suggests bilateral disease or a solitary functioning kidney and should prompt evaluation [1,12]. Additional clues include kidney size asymmetry or a small/atrophic kidney on ultrasound, unexplained declines in renal function during diuresis, and the presence of bruits or diffuse atherosclerosis in other vascular beds [1,11].

Laterality and phenotype

Pickering phenotype is classically tied to bilateral atherosclerotic RAS or unilateral RAS in a solitary functioning kidney, where loss of contralateral natriuresis makes the physiology volume-dependent and highly prone to flash pulmonary oedema [1,3]. In contrast, unilateral RAS with two functioning kidneys is uncommon because the unaffected kidney can offload sodium and water. So when it does present with flash oedema, reports consistently frame it as rare and usually occurs in the context of impaired ventricular function or a small or hypofunctioning stenosed kidney [3,8]. This rarity matters clinically. Establishing laterality and differential renal function helps determine whether renin angiotensin system blockade can be used safely, whether the stenosis is the true driver of this decompensation, and whether the benefit-to-risk balance favours revascularisation in a flash oedema phenotype, or a medical-first approach when the affected kidney is small and salvage is limited [1,3,9,10,12].

Management

In this patient, the episode of flash pulmonary oedema likely reflected a multifactorial process, including long-standing hypertensive heart and kidney disease, with unilateral RAS as a contributor, so a medical-first strategy is favoured. Management of atherosclerotic RAS involves optimising medical therapy and revascularisation. Guidelines support aggressive risk factor control, blood pressure and volume optimisation, lipid lowering, antiplatelet therapy when appropriate, and smoking cessation for all patients [3,9,12,13]. Endovascular stenting is the dominant revascularisation strategy for ostial atherosclerotic lesions, while open surgery is reserved for complex anatomy, aneurysm, or failed endovascular therapy [9,12,14]. Large, randomised trials in broad RAS cohorts showed no overall advantage of routine stenting over medical therapy regarding blood pressure or kidney function, although these trials largely did not include patients with flash pulmonary oedema or clearly haemodynamically severe disease [8,9]. By contrast, expert statements and guidelines support revascularisation as appropriate in selected patients with recurrent, otherwise unexplained pulmonary oedema or refractory heart failure when a haemodynamically significant lesion is present, because relieving the stenosis can reduce pulmonary congestion and stabilise renal function in that phenotype [3,13,14].

Since the stenosis is unilateral and the contralateral kidney is normal, the volume-dependent drive that is present in bilateral or single kidney presentations is reduced, and the affected kidney is small with cortical scarring and reduced differential function, which predicts limited renal salvage after stenting [3,7,9,13]. The patient has shown clinical stability with improved ejection fraction on optimised medical therapy, and blood pressure and congestion have been controlled without recurrent flash events, lowering the immediate need for an invasive approach [3,7]. In this context, the expected gradual benefits of stenting are moderate and must be evaluated against risks including atheroembolism, contrast exposure, restenosis, and access complications [7,9,10]. Therefore, a medical-first plan is reasonable, with continued optimisation of antihypertensive and heart failure therapy, careful monitoring of creatinine and potassium if renin angiotensin system blockade is introduced, and a low threshold to reconsider revascularisation should pulmonary oedema recur, blood pressure remains refractory on best therapy, or renal function decline without another explanation [9,12,13].

What this case adds to the literature

This case shows that flash pulmonary oedema can occur in a patient with unilateral atherosclerotic RAS despite two functioning kidneys, when the culprit kidney is small and hypofunctioning and cardiac compliance is impaired. It illustrates how a renovascular contribution may be considered in this context. This is an uncommon but documented phenotype that extends beyond the classic bilateral or solitary kidney pattern [2,8]. It underscores how this presentation remains under-recognised and easily misattributed to ‘generic’ heart failure, reinforcing the need to keep a renovascular trigger on the radar in hypertensive oedema [3]. It also illustrates decision-making when renal salvage is likely limited: with unilateral disease, a small, scarred kidney, and clinical stability on optimal therapy, a medical-first strategy is defensible, reserving revascularisation for recurrence or refractory control [1,12,13].

Learning points

Unilateral RAS with two kidneys may contribute to or be associated with flash oedema when the stenosed kidney is small and hypofunctioning or when ventricular compliance is reduced [2,8].

In such cases, expected renal salvage is limited, and medical treatment with clear revascularisation rules is appropriate [1,12,13].

Neutral trial results in broad RAS cohorts should not deter targeted evaluation and treatment in the Pickering phenotype [9,10,12,13].

## Conclusions

Flash pulmonary oedema should prompt consideration of a renovascular trigger, even when renal artery disease appears unilateral, particularly in the presence of a small, hypofunctioning kidney. In patients with a small culprit kidney and preserved contralateral function, the expected renal salvage from stenting is often limited, making a medical-first approach appropriate when blood pressure and congestion can be controlled. Revascularisation should remain an option for recurrence, refractory hypertension, or otherwise unexplained renal decline. Early recognition and a selective, phenotype-driven strategy can help balance the risks and benefits of intervention, avoiding both missed opportunities for revascularisation and unnecessary procedures.
